# Platycodin D Suppresses Type 2 Porcine Reproductive and Respiratory Syndrome Virus In Primary and Established Cell Lines

**DOI:** 10.3390/v10110657

**Published:** 2018-11-21

**Authors:** Mingxin Zhang, Taofeng Du, Feixiang Long, Xia Yang, Yankuo Sun, Mubing Duan, Guihong Zhang, Yahong Liu, En-min Zhou, Weisan Chen, Jianxin Chen

**Affiliations:** 1 Guangdong Provincial Key Laboratory of Veterinary Pharmaceutics Development and Safety Evaluation, College of Veterinary Medicine, South China Agricultural University, Guangzhou 510642, China; mxzhang@stu.scau.edu.cn (M.Z.); 20171027008@stu.scau.edu.cn (F.L.); m15902070514@163.com (X.Y.); yankuosun@stu.scau.edu.cn (Y.S.); guihongzh@scau.edu.cn (G.Z.); gale@scau.edu.cn (Y.L.); 2 Experimental Station of Veterinary Pharmacology and Veterinary Biotechnology, Ministry of Agriculture, College of Veterinary Medicine, Northwest A&F University, Yangling 712100, Shaanxi, China; taofengdu@nwafu.edu.cn (T.D.); zhouem@nwsuaf.edu.cn (E.-m.Z.); 3 Department of Biochemistry and Genetics, La Trobe Institute for Molecular Science, La Trobe University, Melbourne, Victoria 3086, Australia; e.duan@latrobe.edu.au

**Keywords:** porcine reproductive and respiratory syndrome virus (PRRSV), platycodin D (PD), antiviral, Marc-145 cells, porcine alveolar macrophage (PAM), cytokine

## Abstract

Porcine reproductive and respiratory syndrome virus (PRRSV) is a continuous threat to the pork industry as it continues to cause significant economic loss worldwide. Currently, vaccination strategies provide very limited protection against PRRSV transmission. Consequently, there is an urgent need to develop new antiviral strategies. Platycodin D (PD) is one of the major bioactive triterpenoid saponins derived from *Platycodon grandiflorum*, a traditional Chinese medicine used as an expectorant for pulmonary diseases and a remedy for respiratory disorders. Here, we demonstrate that PD exhibits potent activity against PRRSV infection in Marc-145 cells and primary porcine alveolar macrophages. PD exhibited broad-spectrum inhibitory activities in vitro against high pathogenic type 2 PRRSV GD-HD strain and GD-XH strain as well as classical CH-1a and VR2332 strains. PD at concentrations ranging 1–4 μM significantly inhibited PRRSV RNA synthesis, viral protein expression and progeny virus production in a dose-dependent manner. EC_50_ values of PD against four tested PRRSV strains infection in Marc-145 cells ranged from 0.74 to 1.76 μM. Mechanistically, PD inhibited PRRSV replication by directly interacting with virions therefore affecting multiple stages of the virus life cycle, including viral entry and progeny virus release. In addition, PD decreased PRRSV- and LPS-induced cytokine (IFN-α, IFN-β, IL-1α, IL-6, IL-8 and TNF-α) production in PAMs. Altogether, our findings suggested that PD is a potent inhibitor of PPRSV infection in vitro. However, further in vivo studies are necessary to confirm PD as a potential novel and effective PPRSV inhibitor in swine.

## 1. Introduction

Porcine reproductive and respiratory syndrome (PRRS) is characterized by reproductive failure in sows, severe respiratory disease and poor growth performance in piglets and growing pigs. Porcine reproductive and respiratory syndrome virus (PRRSV) causes great economic losses to the swine industry worldwide [1,2,3]. This disease was first recognized in 1987 in the United States [4], and subsequently became a pandemic disease in North America, Europe, and Asia within the succeeding years [3,4,5,6]. By now, PRRS has emerged in almost all pork-producing countries. In 2013, the annual loss caused by PRRS to the American swine industry was approximately 664 million USD [7]. In 2006, a highly pathogenic PRRSV (HP-PRRSV) strain with discontinuous 30 amino acid depletion in nsp2 protein associated with porcine high fever syndrome was reported by China, which overwhelmed the swine industries in China and Vietnam [6,8,9].

PRRS is caused by PRRSV, an enveloped, single stranded positive-sense RNA virus that clusters in the order of Nidovirales and the family of *Arteriviridae*. There are two well-known PRRSV species: type 1, or European-like (prototype Lelystad), and type 2, or North American-like (prototype VR-2332) [10]. These two species share approximately 60% sequence identity and exhibit serotype differences [4]. Unlike other members of the genus *Arterivirus*, which exhibit relatively broad cell tropism [11], PRRSV infection is highly restricted to cells of the monocyte-macrophage lineage such as porcine alveolar macrophages (PAMs), the primary targets of PRRSV in vivo [12]. Most importantly, PRRSV is reported to rapidly mutate at an estimated rate of 3.29 × 10^−3^ substitutions per nucleotide site per year and consequently evolves to form new strains frequently [13].

At present, vaccination remains the most prevalent method of controlling PRRSV infections. However, currently commercially available vaccines often fail to provide sufficient protection for infections due to a number of factors associated with virus biology [14]. These include high antigenic heterogeneity and variability, replication in and destruction of lung alveolar macrophages, antibody-dependent enhancement (ADE) and viral persistence [15]. Consequently, PRRSV remains a great challenge for the swine industry [16]. The development of new strategies for controlling this infectious disease, especially novel drugs against PRRSV is an urgent need. According to Traditional Chinese Medicines (TCMs), many natural compounds and herbal components have proven antiviral activities [17], including those against PRRSV, such as flavaspidic acid AB [18] and glycyrrhizin [19]. Despite this, no effective drugs are commercially available for treating PRRSV infections.

**Figure 1:**
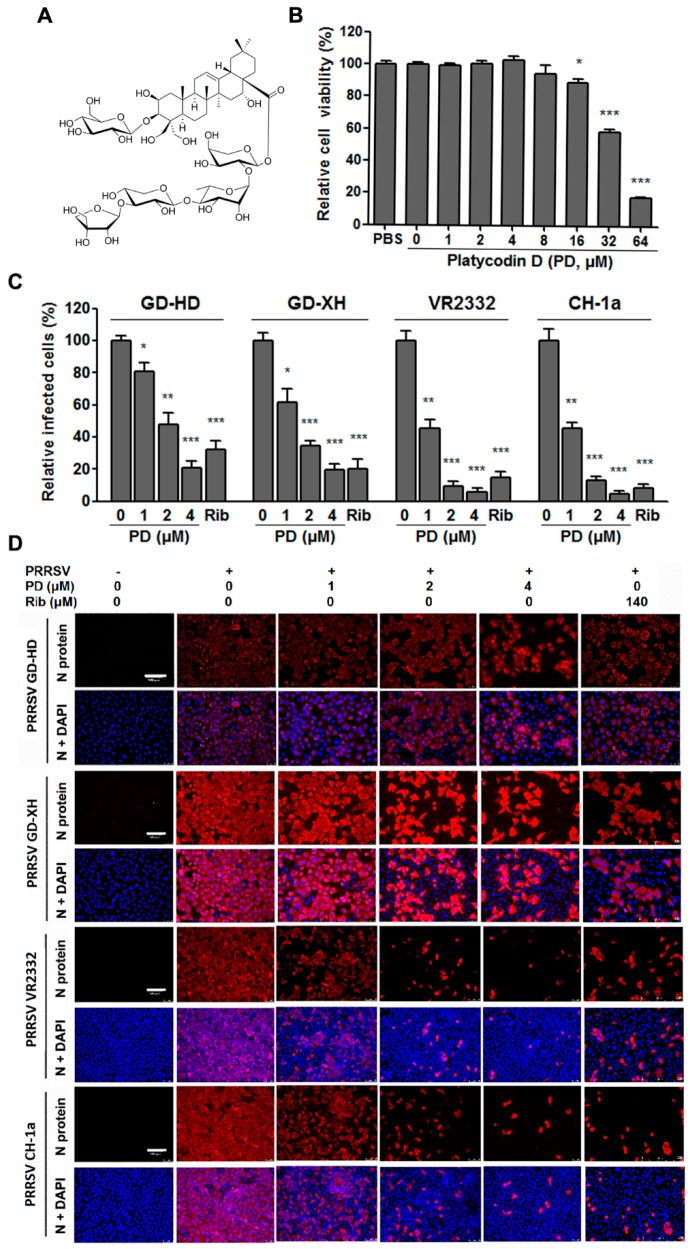
The cellular toxicity and anti-PRRSV activity of PD in MARC-145 cell cultures. (**A**) Chemical structures of platycodin D (PD). (**B**) Cellular toxicity of PD was examined in MARC-145 cells using MTT assay and was expressed as relative cell viability of the viable cells in the absence of the compound (set up as 100%). (**C**,**D**) Antiviral activity of PD against PRRSV strain (GD-HD, GD-XH, VR2332 and CH-1a) infections in MARC-145 cells was examined using immunofluorescence assay (IFA). Cells grown in 96-well plates were infected with PRRSV (0.05 MOI) for 2 h at 37 °C and then cultured in fresh medium containing various concentrations of PD. IFA for the N protein of PRRSV was performed at 48 hpi using Alexa Fluor 568-conjugated goat anti-mouse secondary antibody (red). Nuclei were counterstained using 4,6-diamidino-2-phenylindole (DAPI) (blue). Results shown in **C** are the mean values of percentages of PRRSV-infected cell ratio in PD-treated groups compared to the DMSO-treated control (0 μM PD, set as 100%) from three independent IFA experiments, and **D** is one representative IFA data from **C**. Scale bar: 100 µm. Statistical significances are denoted by * *p* < 0.05, ** *p* < 0.01, and *** *p* < 0.001.

The root of *Platycodon grandiflorum* A. DC (Campanulaceae) is a well-known Chinese herb used as an expectorant for pulmonary diseases and a remedy for respiratory disorders. Interestingly, saponins have been shown to be the main bioactive components of the root of *P. grandiflorum* [20]. Platycodin D (PD), an oleanane type triterpenoid saponin with two sugar chains attaching to position C-3 and C-28 of aglycone (Figure 1A), is regarded as the most biologically potent among platycodin saponins [21]. Previous studies showed that PD has anti-tumor [22], anti-inflammatory [23] and immunological adjuvant activities [20]. PD was also identified to have hepatoprotective and anti-hepatitis C virus (HCV) activities [24]. Here, we demonstrated that PD potently inhibited PRRSV infection in Marc-145 cells and PAMs at micromolar concentrations and in a dose-dependent manner. The mechanisms of PD inhibiting PRRSV were also investigated. To our knowledge, this is the first report of PD’s anti-PPRSV activities.

## 2. Materials and Methods 

### 2.1. Cell Lines and Viruses

Marc-145 cells, a PRRSV-permissive cell line derived from African green monkey kidney cell line MA-104 [25] were obtained from the American Type Culture Collection (ATCC) and grown in Dulbecco’s minimum essential medium (DMEM, Gibco, UT, USA) supplemented with 10% fetal bovine serum (FBS, Biological Industries, Kibbutz Beit Haemek, Israel) and 100 IU/mL of penicillin and 100 μg/mL streptomycin at 37 °C with 5% CO_2_.

Porcine alveolar macrophages (PAMs) were obtained from the lungs of 4- to 6-week-old PRRSV-negative Large-White piglets (Xinli Pig Farm, Wuzhou, China) by lung lavage according to a previously described method [26]. Briefly, the lungs were washed three times with pre-cooled phosphate buffered saline (PBS) solution containing penicillin (300 IU/mL) and streptomycin (300 μg/mL). Cells were centrifuged at 800× *g* for 10 min, resuspended in RPMI 1640 supplemented with 10% FBS and 100 IU/mL of penicillin and 100 μg/mL streptomycin at 1 × 10^6^ cells/mL in 6-well plate, and then incubated at 37 °C for 2 h. The suspending cells (mainly lymphocytes and red blood cells) were removed and adherent cells were PAMs (shown in Figure S1).

Four type 2 PRRSV strains including traditional CH-1a and VR2332 strains, and highly pathogenic GD-HD and GD-XH strains [27] were propagated in Marc-145 cells in DMEM with 3% FBS (essential medium). Virus preparations were titered and stored at −80 °C. Virus titers were determined using a microtitration infectivity assay [28]. Briefly, virus preparations were 10-fold serially diluted in essential medium. Confluent monolayers of Marc-145 cells or PAMs prepared in 96-well plates were inoculated in quadruplicates with 100 μL of each sample and incubated for 2 h at 37 °C. The inoculum was then discarded, and the cell monolayer replenished with fresh essential medium and incubated for an additional 72 h and monitored for cytopathic effects (CPE) daily. The titer of each preparation was calculated based on the amount of CPE and expressed as a 50% tissue culture infective dose (TCID_50_)/1 mL.

### 2.2. Preparation of PD and Chemicals

Platycodin D (PD) was purchased from Chen du Pufei De Biotech Co., Ltd. (Chen du, China), with a purity of ≥99.3%. HPLC and Mass spectrum of PD were shown in Supplementary Figure S2A,B. Ribavirin, a broad-spectrum antiviral agent, was used as positive control and purchased form Star Lake Bioscience Co., Ltd. (Zhaoqing, China). PD and ribavirin were dissolved in dimethyl sulfoxide (DMSO, Sigma-Aldrich, MA, USA) and diluted with essential medium before use. The final concentration of DMSO in the culture medium was less than 0.4%.

### 2.3. Cytotoxicity Assay

The cytotoxicity of PD was evaluated using MTT assay [18]. Briefly, for Marc-145 cells, 5 × 10^4^ cells (per well) were seeded in 96-well plates and grown at 37 °C for 36 h. For PAMs, 2 × 10^5^ cells (per well) were seeded in 96-well plates and incubated at 37 °C for 12 h. The medium was replaced with fresh medium containing serially diluted compounds and the cells were further incubated for 48 h. The culture medium was removed and replaced with 100 μL 3-(4,5-dimethylthiozol-2-yl)-3,5 -dipheryl tetrazolium bromide (MTT; Sigma-Aldrich) solution (0.5 mg/mL in PBS) and incubated at 37 °C for 4 h. After removal of the supernatant, 150 μL of DMSO was added to all of the wells to dissolve the formazan crystals for 10 min at 37 °C. Cell viability was measured as the absorbance at 490 nm with a microplate reader (Thermo fisher scientific, MA, USA) and expressed as a percentage of the control level. The mean optical density (OD) values from six wells per treatment were used as the cell viability index. The 50% cytotoxic concentration (CC_50_) was analyzed by GraphPad Prism 5.0 (GraphPad Software, San Diego, CA, USA).

### 2.4. Antiviral Activity Assay

The antiviral activity assay was performed for compounds to be tested to compare their in vitro capacities in inhibiting PRRSV replication. Marc-145 or PAM cell monolayers grown in 96-well plates were infected with PRRSV (0.05 MOI for Marc-145 cells and 0.5 MOI for PAMs) in essential medium for 2 h at 37 °C. Supernatants were removed and fresh DMEM containing different concentrations of each compound then added. Cells and supernatants were then collected at the indicated time points post-infection and subjected to three freeze-thaw cycles at −80 °C and 4 °C respectively to ensure maximal release of cellular virions. Final supernatant viral titer was determined by the end point dilution assay using Marc-145 cells and expressed as log_10_ TCID_50_/1 mL [29]. 

### 2.5. Indirect Immunofluorescence Assay (IFA)

For immunostaining, the PRRSV-infected or uninfected cells were fixed with 4% paraformaldehyde for 10 min, permeabilized with 0.25% Triton X-100 for 10 min at room temperature (RT), blocked with 1% bovine serum albumin (BSA) for 60 min at RT and then incubated with a mouse monoclonal antibody against the N-protein of PRRSV (clone 4A5, 1:400 dilution, MEDIAN Diagnostics, Korea) at 4 °C overnight. After three washes with PBS, the cells were incubated for 1 h at RT with a goat anti-mouse secondary antibody conjugated with Alexa Fluor^®^ 568 (red) (Cell Signaling Technology, MA, USA) at 1:1000 dilution. Nuclei were counterstained using 50 μL of 4,6-diamidino-2-phenylindole (DAPI, 300 nM; Sigma-Aldrich) (blue). Immunofluorescence was captured using the Leica DMI 4000B fluorescence microscope (Leica, Wetzlar, Germany). Blue and red fluorescence spots were counted as the total and PRRSV-infected cell number respectively in every IFA image. The percentage of infected cell counts among total cell counts was considered as the infection rate. Relative infected-cell percentage was determined by the ratio of the infection rate in PD-treated groups to that in DMSO-treated control. The EC_50_ value (the concentration required to protect 50% cells from PRRSV infection) was determined by plotting the relative infected-cell percentage as a function of compound concentration and calculated with the GraphPad Prism 5.0 software.

### 2.6. Real-Time Reverse-Transcription PCR (RT-PCR)

Total RNA was extracted from cells or culture supernatants using the total RNA rapid extraction kit (Fastagen, Shanghai, China) following manufacturer’s instructions. RNA was reverse-transcribed into first-strand cDNA using a reverse transcription kit (TaKaRa, Dalian, China). PCR amplification was performed on 1 μL of reverse-transcribed product with primers designed against PRRSV-NSP9, cytokines (IFN-α, IFN-β, IL-1α, IL-6, IL-8 and TNF-α) and GAPDH (glyceraldehyde-3-phosphate dehydrogenase, used as the endogenous control). The primers used for PCR amplification are listed in Table 1 [18]. Real-time PCR was performed using 2×RealStar Green Power Mixture (containing SYBR Green I Dye) (Genstar, Beijing, China) on the CFX96 Real-time PCR system (Bio-Rad, CA, USA). Relative mRNA expression were calculated by 2^−ΔΔCT^ method using DMSO-treated infected cells or DMSO-treated mock-infected cells as reference samples for determining PRRSV-NSP9 and cytokine gene expression, respectively [30,31]. To assess the effect of PD on transcriptional activation of cytokines in PRRSV infected cells, the relative fold change of each cytokine gene expression was calculated and compared between virus-infected and mock-infected PAM cells and between PD-treated virus-infected and virus-infected cells. 

### 2.7. Western Blot Analysis

PRRSV-infected or uninfected Marc-145 cells treated with PD were lysed in RIPA lysis buffer containing 1 mM phenylmethylsulfonylfluoride (Beyotime, Haimen, China) at 4 °C. The supernatant was harvested after centrifugation (15,000 g for 30 min at 4 °C) and the total protein for each sample measured using the BCA protein assay kit (Beyotime, China). Ten micrograms of total protein per sample was electrophoresed onto a 12% SDS-PAGE gel and transferred to polyvinylidene-fluoride (PVDF) membranes (Millipore, MA, USA). After blocking, membranes were incubated with a mouse anti-PRRSV N-protein monoclonal antibody (clone 4A5, MEDIAN Diagnostics, Chuncheon, Korea) or a mouse anti-GAPDH monoclonal antibody (GoodHere, Hangzhou, China) at 1:1000 dilution at 4 °C overnight. Anti-mouse IgG (H+L) (DyLight^®^ 800 Conjugate, 1:1000, Cell Signaling Technology) was used as the secondary antibody for 1 h incubation at RT. The Odyssey system (LICOR, CT, USA) was used to analyze the PVDF membranes.

### 2.8. PRRSV Binding Assay

Marc-145 cells were pre-chilled at 4 °C for 1 h and the medium was replaced with DMEM containing PRRSV (0.5 MOI) with or without PD. Cells were incubated for an additional 2 h at 4 °C to facilitate virus binding, followed by three washes with PBS to remove any unbound virus particles and chemicals. Cells were then subjected to RT-PCR for viral mRNA analysis.

### 2.9. PRRSV Internalization Assay

Marc-145 cells were pre-chilled at 4 °C for 1 h and then incubated in essential medium containing PRRSV (0.5 MOI) at 4 °C for 2 h (a time window which facilitates virus binding but not virus internalization). After three washes with PBS, cells were placed in fresh medium with or without PD and shifted to 37 °C for an additional 3 h incubation to facilitate virus internalization. Cells and supernatants were then collected for RT-PCR analysis.

### 2.10. PRRSV RNA Replication Assay

Marc-145 cells were infected with PRRSV (0.05 MOI) for 6 h at 37 °C, and then washed three times with PBS to remove free virus particles. Fresh medium containing 4 μM PD was added and cells were collected at 2 or 4 h after PD addition for RT-PCR analysis.

### 2.11. PRRSV Release Assay

Marc-145 cells were infected with PRRSV (0.05 MOI) for 2 h at 37 °C and then cultured in fresh medium for 24 h. The supernatants were then removed and the cells were cultured in fresh medium with or without PD. At 2 h post medium switching, the culture supernatants and the cells were harvested separately for RT-PCR analysis.

### 2.12. PD Pretreatment on Marc-145 Cells

To investigate whether PD inhibits PRRSV replication through altering host cell susceptibility, Marc-145 cells were pretreated with PD in essential medium for 2 h at 37 °C. After three washes with PBS, cells were infected with PRRSV (0.05 MOI) for 2 h and collected at 48 h post-infection (hpi) for RT-PCR analysis.

### 2.13. Direct PRRSV-PD Interaction

To investigate whether PD directly interact with the virus, 100 µL of PRRSV (1 MOI) or equal dose of UV-inactivated (ultraviolet radiation 30 min) PRRSV was mixed with various concentrations of PD in essential medium (0.9 mL) and incubated for 1 h at 37 °C. Then, PRRSV and PD were separated by ultrafiltration centrifugation. Briefly, the mixture of PRRSV and PD was added to an ultrafiltration tube (0.5 mL, 30 K cutoff) followed by centrifugation (7500× *g*, 10 min) at 4 °C. PRRSV trapped in the ultrafiltration tube were washed twice with essential medium to remove residual PD, and were then resuspended in essential medium for infecting Marc-145 cells grown in 6-well plates for 2 h. After three washes with PBS, the cells were cultured in fresh medium for an additional 48 h at 37 °C and then subjected to viral mRNA analysis using RT-PCR. The filtrates were subjected to PD quantitation analysis using a HPLC-MS/MS system.

### 2.14. HPLC-MS/MS Analysis

Analysis of PD concentration was carried out using an Agilent 1200 series high-performance liquid chromatography (HPLC) system coupled with an Applied Biosystem API 4000 triple quadrupole mass spectrometer. Chromatographic separation was performed using an Agilent Zorbax SB-Aq C18 column (150 mm × 2.1 mm i.d., 3.5 μm). The mobile phase consisted of acetonitrile (A) and 0.1% formic acid in Milli Q water (B) with the following linear gradient elution program: 0.0–1.0 min 5% A; 1.0–5.0 min 5%–90% A; 5.0–7.0 min 90% A; 7.0–7.1 min 90%–5% A; 7.1–15 min 90% A. 

The mass analysis was carried out under the negative electrospray ionization mode. The optimum conditions of multiple reaction monitoring (MRM) were carried out at the following parameters: ion spray voltage (IS), −4500 V; ion source gas (GS1 and GS2), 65 and 65 psi, respectively. The transitions of *m*/*z* 1223.9→469.3 was used for quantification, and of *m*/*z* 1223.9→681.5 was used for identification (Figure S2).

### 2.15. Cell-to-Cell Spreading Assay

Cell-to-cell spreading assay was performed as previously described [20]. Briefly, Marc-145 cells were incubated with PRRSV at 37 °C for 3 h in the absence of polyclonal PRRSV-neutralizing serum to permit viral internalization, or in the presence of the serum to neutralize virions and prevent infection. Free virus particles were then removed and cells were cultured for 48 h in medium containing the neutralizing serum and various concentrations of PD. Cells cultured in medium with 0.4% DMSO served as a control. The infected cells were observed using indirect immunofluorescent staining of the PRRSV N protein.

### 2.16. Statistical Analysis

All experiments were performed at least three times. The results were presented as mean ± standard deviation (SD). Statistical significance was determined by Student’s *t* test when only two groups were compared or by one-way analysis of variance (ANOVA) when more than two groups were compared. * *p* < 0.05, ** *p* < 0.01, and *** *p* < 0.001 were considered to be statistically significant at different levels.

## 3. Results

### 3.1. PD Inhibits PRRSV Infection in MARC-145 Cells

We first tested the cytotoxicity of PD (Figure 1A) to MARC-145 cells, which are permissive for PRRSV infection in vitro, by MTT assay. As shown in Figure 1B, PD did not impair MARC-145 cell viability at even 8 Μm. However, at concentrations from 16 To 64 Μm, PD exhibited significant and dose-dependent cytotoxic effects on MARC-145 cells. The 50% cytotoxic concentration (CC_50_) of PD on MARC-145 Cells was 36.2 Μm.

Next, we examined the antiviral effects of PD against PRRSV strains (GD-HD, GD-XH, VR2333 and CH-1a) using MARC-145 cells and immunofluorescence microscopy at 48 hpi. As shown in Figure 1C,D, PRRSV infection was significantly inhibited by PD in a dose-dependent manner. The 50% effective concentrations (EC_50_) of PD against the four PRRSV strain infections were determined to range from 0.74 to 1.76 μM by counting infected cells from IFA images, and corresponding selectivity index (SI) ranged from 49 to 21 (Table 2). These results indicated that PD has potent inhibition against PRRSV infections and this effect is strain independent.

**Figure 2:**
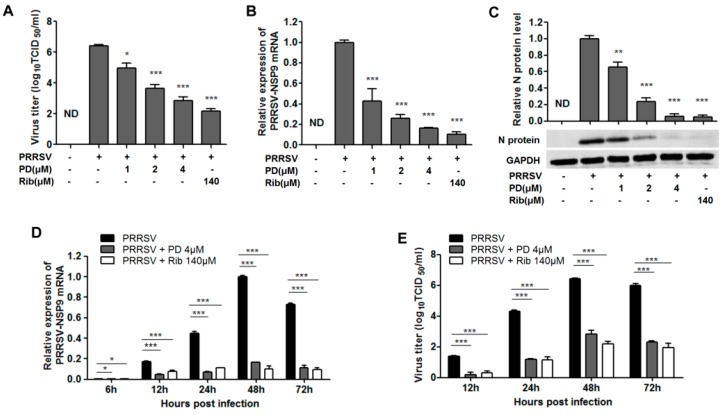
Confirming PD’s anti-PRRSV activity in PRRSV-infected MARC-145 cells. Cells grown in 6-well plates were infected with PRRSV GD-HD (0.05 MOI) for 2 h at 37 °C and then cultured in fresh medium containing various concentrations of PD. At 48 h (**A**–**C**) or indicated time-points (**D**,**E**) post infection, the samples were subjected to viral titer, or RT-PCR, or Western blotting analysis. (**A**,**E**) The PRRSV titer was determined after treatment with PD for 48 h (**A**) or indicated time-points (**E**) using the end point dilution assay and expressed as log_10_ TCID_50_/1 mL. (**B**,**D**) Relative PRRSV NSP9 mRNA level was analyzed using real-time RT-PCR at 48 h (**B**) or indicated time-points (**D**) after treatment with PD. Expression of GAPDH was shown as a loading control, and DMSO-treated sample (0 μM PD) at 48 h was used as treatment (or solvent) control (set as 1). (**C**) Expression of viral N protein in cells treated with various concentrations of PD for 48 h was detected by Western blotting. Upper panel: the mean values from three independent experiments. Lower panel: one representative Western blotting image out of three independent experiments. Results shown in upper panel of **C** are normalized N protein levels based on the optical densities (OD) of the bands from three independent experiments. Software Image J was used to analyze band OD; Results from PD treated samples were compared to those from DMSO-treated control groups (0 μM PD) (set as 1). Statistical significances are denoted by * *p* < 0.05, ** *p* < 0.01, and *** *p* < 0.001.

We further examined the antiviral effects of PD on the GD-HD strain using virus titration, RT-PCR and Western-blotting at 48 hpi. As shown in Figure 2A, treatment with PD resulted in a significant reduction of PRRSV titer in a dose-dependent manner. Treatment with 4 μM of PD lead to a 3.6 log reduction in progeny virus production compared to that in DMSO-treated control (Figure 2A). In fact, PD at concentrations between 1 and 4 μM significantly inhibited PRRSV NSP9 RNA levels and N protein levels in a dose-dependent manner in Marc-145 cells (Figure 2B,C). Ribavirin, a well-known inhibitor of viral RNA polymerase, was used as a positive antiviral drug control in this study. Our results showed that 140 μM of ribavirin exhibited a significant inhibition on PRRSV infection in the same assays.

We further studied the PRRSV inhibition kinetics by PD at 4 μM. For the PRRSV-infected control, at 6 hpi the viral RNA level was negligible. The viral mRNA levels increased from 12 hpi to 48 hpi, and then decreased at 72 hpi (Figure 2D). As expected, virus titers exhibited a similar profile at these time-points, as shown in Figure 2E. The addition of 4 μM of PD significantly inhibited viral RNA levels and progeny virus titers at all time-points (Figure 2D,E). 

### 3.2. PD Inhibits PRRSV Infection in PAMs

**Figure 3:**
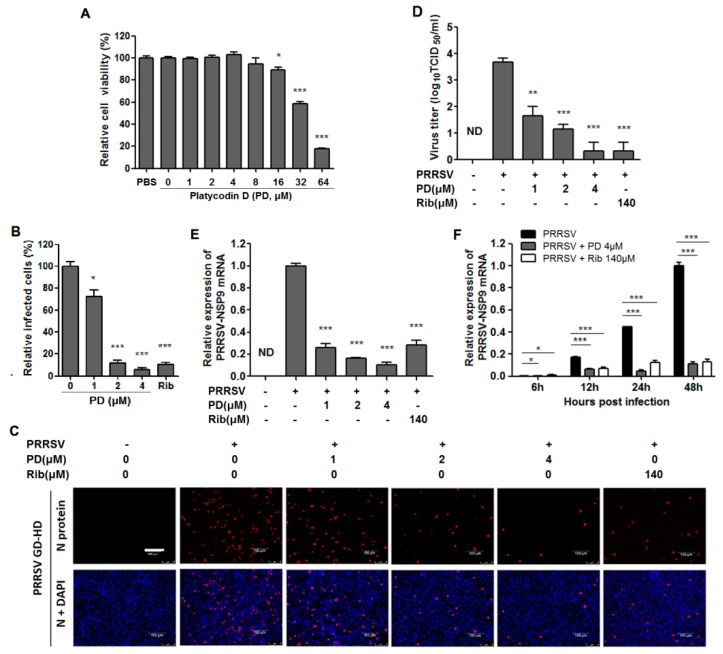
The anti-PRRSV activity and cellular toxicity of PD in PAM cultures. (**A**) Cellular toxicity of PD was examined in PAM cells using an MTT assay as described in the methods. (**B**–**F**) PAMs grown in 96-well plates (**B**,**C**) or 6-well plates (**D**–**F**) were infected with PRRSV GD-HD (0.5 MOI) for 2 h at 37 °C and then treated with PD at various concentrations. Twenty-four hours or indicated time later, the samples were studied by IFA, or viral titer or RT-PCR analysis. (**B**,**C**) IFA for the N protein of PRRSV was performed at 24 hpi using Alexa Fluor 568-conjugated goat anti-mouse antibody as the secondary antibody (red), and nuclei were stained with DAPI (blue). Scale bar: 100 µm. Results shown in (**B**) are the mean values of percentages of PRRSV-infected cell ratio in PD-treated groups compared to the DMSO-treated control (0 μM PD, set as 100%) from three independent IFA experiments, and (**C**) is one representative IFA data from (**B**). (**D**) The PRRSV titer was determined after treatment with PD for 24 h using the end point dilution assay and expressed as log_10_ TCID_50_/1 mL. (**E**,**F**) Relative PRRSV NSP9 mRNA expression of PD treated groups to DMSO-treated control (set as 1) was analyzed using real-time RT-PCR at 24 h (**E**) or indicated time-points (**F**) post PD treatment. * *p* < 0.05, ** *p* < 0.01, and *** *p* < 0.001 compared to DMSO-treated control.

Since PD exerted potent antiviral activity against PRRSV infection in MARC-145 cells, we questioned whether PD was also able to inhibit PRRSV replication in ex vivo PAMs, the major target cell type of PRRSV infection in pigs in vivo. We initially assessed PD cytotoxicity on PAMs using an MTT assay. As shown in Figure 3A, PD exhibited a similar cytotoxicity profile on PAMs to that on MARC-145 cells, and its CC_50_ on PAMs was 35.1 μM. Next, we evaluated the antiviral effects of PD against PRRSV GD-HD infection in PAMs using immunofluorescence microscopy, virus titration and RT-PCR at 24 hpi. As shown in Figure 3B,C, PD significantly reduced PRRSV N-protein levels and showed a dose-dependent PRRSV suppression. A significant reduction of PRRSV titer in a dose-dependent manner was also observed when PRRSV-infected PAMs were treated with PD (Figure 3D). Treatment with 4 μM of PD resulted in a 3.3 log reduction in progeny virus production when compared to that in the DMSO control (Figure 3D). A similar pattern in relative viral mRNA level was also confirmed by RT-PCR analysis following PD treatment, as shown in Figure 3E. A consistent inhibition of PD at 4 μM on GD-HD replication in PAMs from 6 to 48 hpi was also observed by RT-PCR analysis, as shown in Figure 3F. Taken together, PD also effectively inhibited PRRSV infection in ex vivo PAMs.

To explore whether PD possessed the same broad inhibition on other three PRRSV strains (GD-XH, VR2332 and CH-1a) in PAM cultures as it did in MARC-145 cell cultures, viral mRNA expressions at 24 hpi in DMSO- or PD-treated groups were investigated using RT-PCR analysis. As shown in Figure S3, PD treatment significantly reduced viral mRNA expressions in a dose-dependent manner against all three PRRSV strains, similar to the inhibition observed on GD-HD strain.

### 3.3. PD Blocks Attachment and Internalization of PRRSV

**Figure 4:**
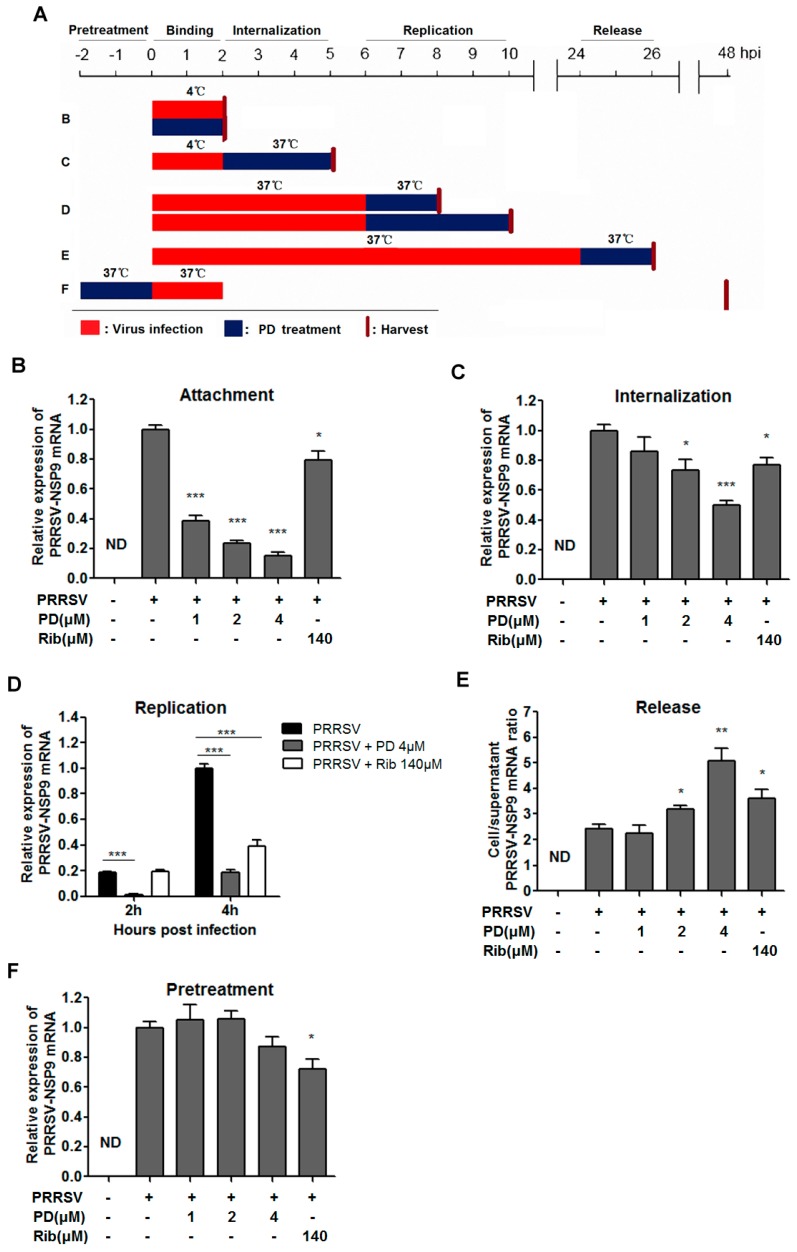
PD inhibits PRRSV entry, replication and release. MARC-145 cells were infected with the PRRSV GD-HD strain at 0.5 or 0.05 MOI. The infected cells were cultured in the presence of various PD concentrations and collected at the indicated time-points post infection to determine viral NSP9 mRNA level by RT-PCR. Cellular GAPDH mRNA was used as a loading control and DMSO-treated sample (set as 1) was used as a reference control. (**A**) Different PD treatment schemes. Red bars represent PRRSV infection period, blue bars represent PD treatment period, and red vertical bars represent end of treatments and cell harvesting. (**B**) Viral binding was investigated following treatment B; (**C**) Viral internalization was investigated following treatment C; (**D**) Viral replication following treatment D; (**E**) Viral release following treatment E; and (**F**) PD pretreatment was performed by treatment F. For B and C, 0.5 MOI PRRSV were used to infect cells; For **D** and **E** and **F**, 0.05 MOI PRRSV were used to infect cells. Statistical significances are denoted by * *p* < 0.05, ** *p* < 0.01, and *** *p* < 0.001.

In order to explore the mechanism of PD-mediated PRRSV inhibition, we first examined the effects of PD on virus entry through cell attachment and subsequent internalization. Marc-145 cells were infected with a higher dose of PRRSV GD-HD (0.5 MOI) in the presence or absence of PD at 4 °C which allows virus binding but not cellular internalization (Figure 4A, treatment B). As shown in Figure 4B, PD treatment at 1, 2 and 4 μM significantly reduced viral mRNA levels in a dose-dependent manner, suggesting that PD directly exerted inhibitory effects on PRRSV binding to MARC-145 cells.

Kreutz and Nauwynck have previously reported that PRRSV is internalized from the surface of MARC-145 cells within 3 hpi [32]. Thus, to examine whether PD might also affect the internalization of PRRSV, PRRSV infected MARC-145 cells were treated with PD during 2–5 hpi (Figure 4A, treatment C). As shown in Figure 4C, virus replication was significantly inhibited when infected cells were treated with 2 and 4 μM of PD, suggesting that PD also inhibited PRRSV internalization. Judging from the viral RNA levels shown in Figure 4B,C, it could be concluded that PD more effectively suppressed PRRSV’s cell attachment than its internalization in cultured MARC-145 cells.

### 3.4. PD Inhibits Viral RNA Replication and Blocks Progeny Virus Release 

MARC-145 cells were infected at 0.05 MOI of PRRSV GD-HD for 6 h at 37 °C to allow normal virus replication and assembly. The infected cells were then cultured in fresh medium containing 4 μM of PD to see whether virus replication was affected. Cells were collected at 8 and 10 hpi and subjected to RT-PCR analysis to assess viral RNA contents (Figure 4A, treatment D). As shown in Figure 4D, PD treatment significantly reduced the viral RNA levels, suggesting that PD inhibited PRRSV RNA replication. 

We wondered whether PD could also affect PRRSV release. Previous studies have demonstrated that PRRSV progeny viruses are released by 8 hpi [19,32]. Our results of the virus proliferation dynamics showed that viral mRNA level and titer increased from 12 hpi to 48 hpi (Figure 2D,E). To explore whether PD directly inhibits viral release, MARC-145 cells were infected with 0.05 MOI of PRRSV GD-HD for 24 h at 37 °C and the cells were then cultured in fresh medium containing PD at serial concentrations for another 2 h. Subsequently, the cells and their culture supernatants were collected separately and the NSP9 RNA were quantified by RT-PCR (Figure 4A, treatment E). As shown in Figure 4E, progeny virus release (viral RNA in the supernatant) is inversely proportional to the ratio of cellular and supernatant viral RNA levels. A lower cell/supernatant viral RNA ratio corresponded to more progeny virus release. Consequently, the addition of 2 and 4 μM of PD was found to significantly inhibit PRRSV virus release from MARC-145 cells.

### 3.5. Pre-Treatment of PD Does Not Affect MARC-145 Cell Susceptibility to PRRSV

To investigate whether PD pretreatment of MARC-145 cells could potentially affect the cell’s susceptibility to PRRSV, serial PD concentrations were added directly to MARC-145 cells for 2 h at 37 °C before PRRSV infection (Figure 4A, treatment F). As shown in Figure 4F, the pre-treatment of MARC-145 cells with PD did not reduce viral RNA levels, suggesting that PD does not directly affect MARC-145 cell’s susceptibility to PRRSV.

### 3.6. PD Directly Interacts with PRRSV

**Figure 5:**
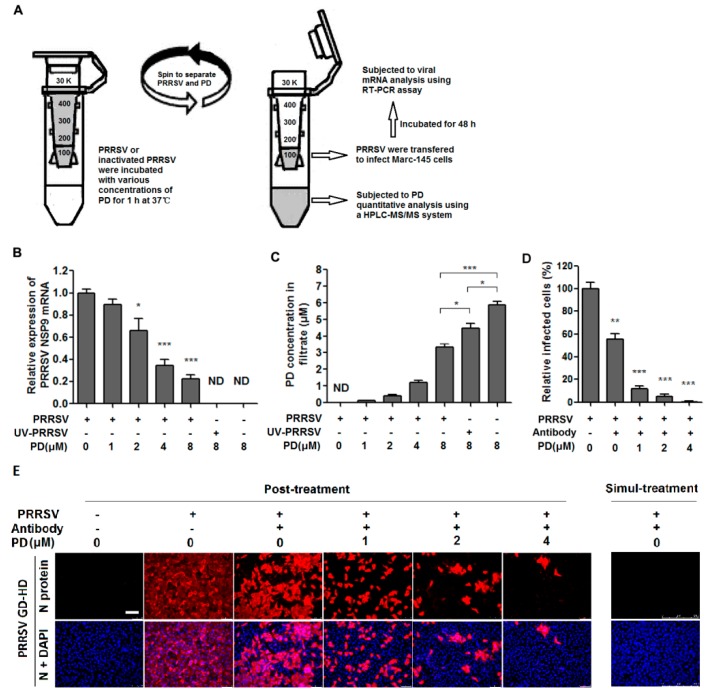
PD interacts with PRRSV directly and inhibits its cell-to-cell spreading. (**A**–**C**) Direct interaction assay of PD and PRRSV. 100 μL of PRRSV GD-HD (2 × 10^6^ PFU) or equal UV-inactivated (120 μW/cm^2^, 30 min) PRRSV GD-HD was mixed with various concentrations of PD in essential medium (0.9 mL total volume) for 1 h at 37 °C. Then PRRSV and PD were separated by ultrafiltration, as shown in (**A**). Recovered PRRSV were resuspended to infect MARC-145 cells. The PD in filtrates was quantified using a HPLC-MS/MS system as described in the methods. The results of relative expression of PRRSV NSP9 mRNA are shown in (**B**), and the results of PD concentration in the filtrates are shown in (**C**). Statistical significances in B and C are denoted by * *p* < 0.05, ** *p* < 0.01, and *** *p* < 0.001. (**D**,**E**) MARC-145 cells grown in 6-well plates were infected with PRRSV (0.05 MOI) at 37 °C for 3 h in the absence of neutralizing serum (post-treatment) to permit viral internalization, or in the presence of neutralizing serum (1:200 dilution, simul-treatment) to neutralize free virions and prevent infection (for post-treatment, neutralizing serum was added at 3 hpi; for simul-treatment, neutralizing antibody added to the culture simultaneously with virus addition). After removing free virus particles, infected cells were cultured for further 48 h in medium containing PD at various concentrations and neutralizing serum (1:200 dilution). IFA for the N protein was performed at 48 hpi as described in the legend to Figure 1 and the methods. Scale bar: 100 µm. Results shown in **D** are the mean values of percentages of PRRSV-infected cell ratio in PD-treated groups compared to the DMSO-treated control (0 μM PD, set as 100%) from three independent IFA experiments, and **E** is one representative IFA data from **E**.

As shown above, PD was able to inhibit PRRSV at every stage of its life cycle, which lead us to wonder whether PD was able to directly interact with PRRSV. We therefore mixed the virus with PD at various concentrations in essential medium for 1 h at 37 °C, and then separated PRRSV from PD via ultrafiltration as shown in Figure 5A. PRRSV left in the ultrafiltration were resuspended in essential medium and used for infecting MARC-145 cells and subsequent viral mRNA analysis using RT-PCR. The filtrates were subjected to PD quantitative analysis using a HPLC-MS/MS system. We also added UV-inactivated PRRSV to see whether UV-inactivation might be able to influence such interaction. As shown in Figure 5B, co-incubation of PD (2, 4 and 8 μM) with virus significantly weakened the ability of PRRSV to infect MARC-145 cells in a dose-dependent manner, demonstrating that PD did directly interact with PRRSV particles. 

In the filtrate derived from the initial PD preparation (8 μM), the ultrafiltration process resulted in a partial PD loss as only 5.92 μM of PD was recovered (Figure 5C). However, PD and PRRSV co-incubation lead to only recovery of 3.36 μM of PD (Figure 5C). Importantly, more PD (4.48 μM) was recovered from the co-incubation of PD with equal amount of UV-inactivated PRRSV, indicating the PD-PRRSV direct interaction was negatively affected by UV-inactivation. 

### 3.7. PD Inhibits Cell-to-Cell PRRSV Spreading in Cell Culture

Not only can viruses infect cells by a cell-free mechanism, viruses can also spread directly to adjacent cells without passing a cell-free stage. Thus, we next examined whether PD could block the cell-to-cell spreading of PRRSV. To rule out the possibility of the extracellular spreading of viruses, PRRSV-neutralizing serum was added in the culture medium. As shown in Figure 5E, when neutralizing antibodies and PRRSV were simultaneously added to the cells (Simul-treatment), virus replication was completely inhibited, indicating effective PRRSV neutralization by the antibodies in medium. While neutralizing antibodies were added at 3 hpi (Post-treatment), many cells were still infected although a reduction of virus-infected cells was observed compared with the control, indicating that the extracellular viral spreading was attenuated. When PD was added, the size of the PRRSV-infected foci was decreased in a dose-dependent manner (Figure 5D,E). At higher concentration (4 μM), PD could reduce the foci to 1/300 compared to the control (Figure 5D,E). These data demonstrated that PD could inhibit PRRSV infection by blocking the cell-to-cell transmission pathway.

### 3.8. PD Treatment Reduces Cytokine Gene Expression by PRRSV-Infected PAMs

**Figure 6:**
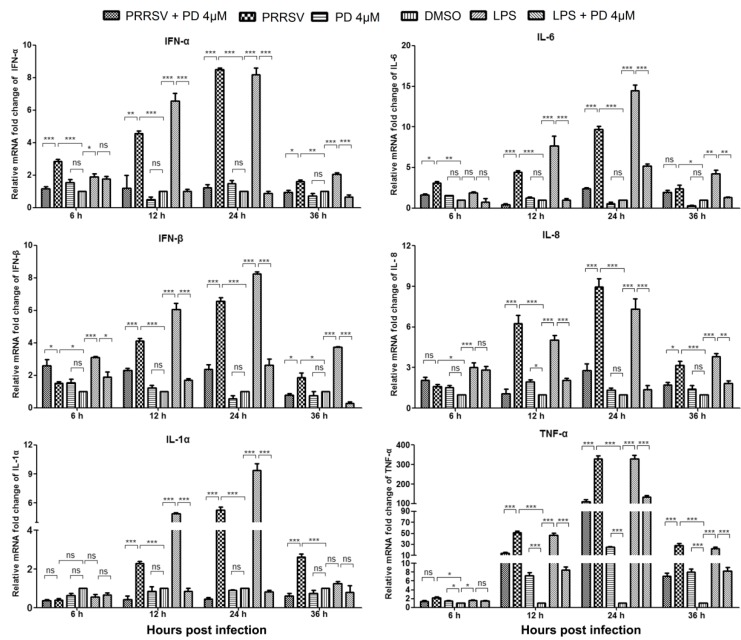
Effect of PD on PRRSV- or LPS-induced cytokine gene expression in PAMs. PAMs were infected with PRRSV GD-HD (0.5 MOI) for 2 h at 37 °C and then cultured in fresh medium in the presence or absence of 4 μM PD. As a parallel positive control, 100 ng/mL of LPS were added to PAMs to stimulate cytokine production. The mRNA level of each cytokine was assessed by RT-PCR. Relative expression (fold change) in comparison with DMSO-treated cells (mock-infected, set as 1) is shown. The data represent the mean values from three independent experiments. Statistical significances are denoted by * *p* < 0.05, ** *p* < 0.01, and *** *p* < 0.001, ns means no significant difference.

PRRSV infection induces the release of pro-inflammatory and antiviral cytokines, which influence the pathological outcome of the infection. To investigate whether PD treatment could affect cytokine expression, the expressions of six cytokines including IFN-α, IFN-β, IL-1α, IL-6, IL-8 and TNF-α, known to be involved in antiviral response and inflammation, were analyzed in the presence or absence of PD infection. PAMs were incubated with or without 4 μM PD for 6 h, 12 h, 24 h or 36 h post PRRSV GD-HD infection, and then RT-PCR was performed to assess the relative cytokine mRNA level in the infected PAMs. To show whether PD affects cytokine productions directly through interacting with PRRSV or indirectly through cellular processes, 100 ng/mL of LPS was added to PAMs to stimulate cytokine production not related to PRRSV infection. As shown in Figure 6, PRRSV infection and LPS treatment both elicited remarkable TNF-α RNA expression at 12, 24 and 36 hpi, although much less so for the other five cytokines (IFN-α, IFN-β, IL-1α, IL-6 and IL-8). From 6 to 24 hpi, the RNA expression levels of the six cytokines gradually increased before decreasing at 36 hpi. Interestingly, treatment with PD remarkably reduced all six cytokine expression induced by both PRRSV infection and LPS treatment. It is worth noting that PD treatment induced a significant increase of TNF-α expression in mock-infected PAMs throughout the monitoring period but did not alter RNA expressions of other five cytokines, indicating that PD has a dual-role in regulating TNF-α production.

## 4. Discussion

PRRSV primarily infects PAMs and is characterized by a high rate of gene mutation and recombination [33]. PRRSV-vaccinated or convalescent animals are protected against reinfection in largely a homologous, rather than heterologous, PRRSV strain-specific manner. Furthermore, it is well illustrated that the protective immunity could not clear the rapidly replicating PRRSV in vivo [34,35]. These characteristics of PRRSV have been limiting the efficiencies of commercially available vaccines. Consequently, research aimed at developing new antiviral strategies against PRRSV infection is urgently needed. Traditional Chinese Medicines (TCMs) and natural herbal products often harbor many bioactive compounds, in particular triterpene saponins, which possess anti-inflammatory, antiviral, antibacterial, antioxidant, anti-cancer and immunomodulatory activities. In this study, our findings revealed that platycodin D (PD), one of the major triterpene saponins of the TCM herb *Platycodon grandiflorum*, exhibited potent antiviral activity against PRRSV infection and replication in vitro. PD treatment resulted in remarkable decrease of progeny viruses both in MARC-145 cells and PAMs at concentrations well below its detectable cell cytotoxicity threshold. Our study also used ribavirin, a broad-spectrum anti-viral agent, as a positive control as Amina and co-workers have previously demonstrated its PRRSV suppressing effect in Marc-145 cells [36]. In our study, 4 μM of PD exhibited similar antiviral activity to that of 140 µM of ribavirin against PRRSV in vitro.

PRRSV infects cells via receptor-mediated endocytosis [37,38], in which virus attachment, membrane fusion, and internalization occur sequentially. We first demonstrated that PD impaired PRRSV attachment as PRRSV infection was significantly affected when PD was provided during the virus-cell co-incubation at 4 °C (Figure 4B). We further showed that PD inhibited virus internalization, although the inhibitory effect might be less obvious when compared to that on viral attachment (Figure 4C), viral RNA replication and progeny virus release (Figure 4D,E). We wondered whether these effects were mediated by PD-induced decrease of cell susceptibility to PRRSV, and found that PD pre-treatment did not affect cell susceptibility to PRRSV (Figure 4F). These results suggest that the early stage antiviral effects of PD may be mediated through direct PD–PRRSV interaction.

Indeed, when PD and PRRSV were directly mixed and then separated by ultrafiltration centrifugation, direct interaction was confirmed as PD was found with the recovered PRRSV particles (Figure 5). However, the PD binding site on PRRSV remains unclear. As PD could inhibit PRRSV attachment to the MARC-145 cells, it is most likely that PD binds to one of the PRRSV surface molecules, including E and GP2 to GP5, directly affecting PRRSV binding to its entry receptor including heparan sulphate, sialoadhesin, CD163 and others [39]. Moreover, as we have shown that PD was able to inhibit PRRSV during multiple stages of the virus life cycle, such as RNA replication and virion release, we cannot exclude the possibility that PD may also directly interact with other viral and cellular proteins. For example, Kim and co-workers demonstrated that triterpenoid extract (containing PD and its homologous compounds) from the root of *Platycodon grandiflorum* exhibited inhibitory activity against hepatitis C virus (HCV) RNA replication in HCV subgenomic replicon cells and HCV infected cells by inhibiting NS5B polymerase activity [40]. In this study, PD treatment significantly enhanced TNF-α expression in un-infected PAMs indicating that PD could interact with cellular protein(s) involved in TNF-α regulation, which might endow PD with indirect PRRSV inhibition effect.

PRRSV preferentially replicates in lung macrophages and monocytes [41,42]. The pro-inflammatory cytokines, TNF-α, IL-1, IL-6 and IL-8, are among the first cytokines produced by the alveolar macrophages and monocytes during PRRSV infection. Increased levels of these cytokines in the circulation are responsible for acute systemic inflammation [43]. Conversely, the production of type I interferons (IFNs) IFN-α and IFN-β by virus-infected cells is one of the most effective innate anti-viral immune responses [44]. PRRSV has evolved mechanisms to inhibit type I IFN response to evade host immune surveillance [45]. Here we monitored dynamic changes of six cytokines induced by infection of PRRSV GD-HD, a high pathogenic strain. PRRSV infection elicited remarkable increases of TNF-α expression, but less so for other five cytokines (IFN-α, IFN-β, IL-1α, IL-6 and IL-8) (Figure 6). Such cytokine expression patterns are consistent with observations made by Kang and Lee, in which an immortalized PAM cell line was infected with PRRSV strain VR2332 [46]. In our study, treatment with PD robustly reduced infection-induced expression of six cytokines from 6 hpi to 36 hpi. 

Notably, PD treatment lead to significant TNF-α RNA expression in un-infected PAMs although not the case for other five cytokines studied. Although TNF-α is generally regarded as a pro-inflammatory cytokine, its pleiotropic antiviral functions, including promoting antiviral state in neighboring uninfected cells, recruiting lymphocytes to infection site, selective cytolysis of virus-infected cells, and modulating cell apoptosis/survival, have been well known [47,48]. In fact, addition of recombinant porcine TNF-α significantly reduces PRRSV replication [49]. Interestingly, our results showed a dual-role for PD in regulating TNF-α production: it promoted TNF-α secretion in uninfected PAMs yet inhibited high expression in PRRSV-infected PAMs, indicating that PD might prepare uninfected cells for PRRSV inhibition on one hand and help the infected cells to avoid TNF-α toxicity on the other.

Patricia and co-workers investigated the dynamic cytokine changes in pigs infected with a high- or low-pathogenic genotype 1 PRRSV strain (high-pathogenic Lena and low-pathogenic Finistere strains). From 8 to 15 days post-infection (dpi), Lena-infection induced higher TNF-α and IL-1β detected in the bronchoalveolar lavage fluid (BALF) and blood compared to those induced by Finistere-infection. Similar results were obtained for IL-8 in BALF. At 4 dpi, high levels of serum IFN-α were detected after Lena-infection but not after Finistere-infection. Their results showed that serum levels of TNF-α and IFN-α were correlated with hyperthermia, while BALF levels of IL-1β or IL-8 were strongly correlated to clinical score in the Lena-infection group [50]. In our study, PD treatment exhibited potently inhibition on disordered cytokine expressions induced by PRRSV infection in PAMs, indicating its likely anti-inflammatory effects in PRRSV infected pigs clinically.

It should be noted that the decreased cytokine production might be a direct result of PD’s anti-inflammatory activity or an indirect result of PD’s anti-viral activity or both. To resolve these possibilities, we investigated whether PD was able to inhibit cytokine expression in PAMs induced by LPS in a parallel experiment. The results showed that PD was as capable of inhibiting LPS-induced cytokine expression as inhibiting cytokine production induced by PRRSV infection, indicating its cytokine inhibitory role is not PRRSV-specific. This result is consistent with the observation made by Tao and co-workers, in which PD attenuated LPS-induced acute lung injury in mice [51]. It is worth noting that PRRSV infections are usually accompanied by secondary bacterial infection and abundant TNF-α in the lung, a significant part of the respiratory syndrome [52,53]. PD’s potent inhibition on TNF-α production induced by PRRSV infection or LPS indicates that PD could attenuate pneumonia caused by PRRSV infection and accompanied secondary bacterial infection.

Yang and co-workers verified that PRRSV was able to spread directly to adjacent cells in MARC-145 cell cultures. Consistent with this observation, we confirmed that PRRSV was able to spread in MARC-145 cells efficiently in the presence of neutralizing antibody. PD treatment blocked this transmission in a dose-dependent manner. This effect is likely related to both PD’s direct and indirect effects on PRRSV as discussed above.

In conclusion, our findings demonstrate that PD is a potent inhibitor of PPRSV infections in vitro with characteristics of direct interaction with PRRSV virions, and a likely indirect effect on viral replication and pro-inflammatory cytokine expression. It may be particularly advantageous to use PD as an anti-PRRSV agent, especially considering that the plant source of PD is abundant and PD extraction is convenient. Further in vivo studies will be necessary to confirm PD as a novel and effective PPRSV inhibitor in swine.

## Figures and Tables

**Table 1:** Real-time PCR primer sequences.

Name ^a^	Sequences 5′ to 3′
NSP9-F	5′- CTAAGAGAGGTGGCCTGTCG -3′
NSP9-R	5′- GAGACTCGGCATACAGCACA -3′
GAPDH-F	5′- GCAAAGACTGAACCCACTAATTT -3′
GAPDH-R	5′- TTGCCTCTGTTGTTACTTGGAGAT -3′
IFN-α-F	5′- AGAGCCTCCTGCACCAGTTCT -3
IFN-α-R	5′- TCACTCCTTCTTCCTG -3′
IFN-β-F	5′- AGCACTGGCTGGAATGAAACCG -3′
IFN-β-R	5′- CTCCAGGTCATCCATCTGCCCA -3′
IL-1α-F	5′- CGATGCCCAGCTGTCTTCCCT -3′
IL-1α-R	5′- CGATGCCCAGCTGTCTTCCCT -3′
IL-6-F	5′- TGCCGGCCTGCTGGATAAGC -3′
IL-6-R	5′- TGGCCCTCAGGCTGAACTGC -3′
IL-8-F	5′- CACTGTGAAAATTCAGAAATCATTGTTA -3′
IL-8-R	5′- CTTCACAAATACCTGCACAACCTTC -3′
TNF-α-F	5′- GCTGGGTGCCAAGGACAGAGG -3′
TNF-α-R	5′- TGGTGGTGCCGACAGATGG -3′

^a^ F: forward primer; R: reverse primer.

**Table 2:** Inhibitory activity of platycodin D (PD) against PRRSV infection in MARC-145 cells.

	PRRSV Strain			
	GD-HD	GD-XH	VR2332	CH-1a
^a^ EC_50_ (μM)	1.76 ± 0.40	1.30 ± 0.34	0.80 ± 0.29	0.74 ± 0.25
^b^ Selectivity index (SI)	21	27	45	49

^a^ EC_50_, the concentration required to protect 50% cells from PRRSV infection by counting infected cells from IFA images, as described in the methods. ^b^ SI (selectivity index) is the ratio of CC_50_ to EC_50_. CC_50_ (the 50% cytotoxic concentration of PD on Marc-145 cells) was 36.2 μM. Data were presented as means ± SD of results from three independent experiments.

## Supplementary Materials

The following are available online at http://www.mdpi.com/1999-4915/10/11/657/s1, Figure S1: Micrographs of PAMs from bronch alveolar lung fluid of piglets at 2 h post incubation, Figure S2: HPLC-MS/MS chromatography and mass spectra of PD in filtrate after direct interaction with PRRSV, Figure S3: The anti-PRRSV activity of PD in PAM cultures is strain independent.
