# Supplementary material for: Platycodin D Suppresses Type 2 Porcine Reproductive and Respiratory Syndrome Virus In Primary and Established Cell Lines
Source: Viruses. 2018 Nov 21;10(11):657. doi: 10.3390/v10110657 (PMC6266211; doi:10.3390/v10110657)
Supplement: Supplementary file 1 [file viruses-10-00657-s001.pdf]

Supplementary Materials:

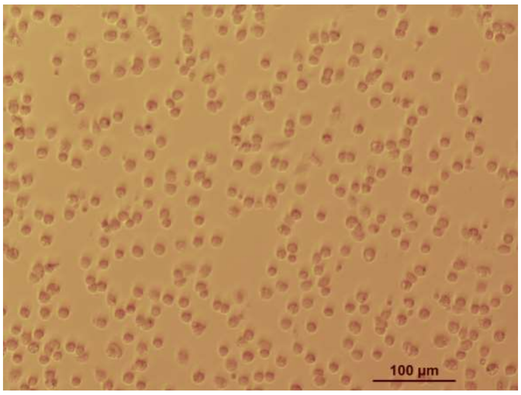

Figure S1. Micrographs of PAMs from bronch alveolar lung fluid of piglets at 2 h post incubation.

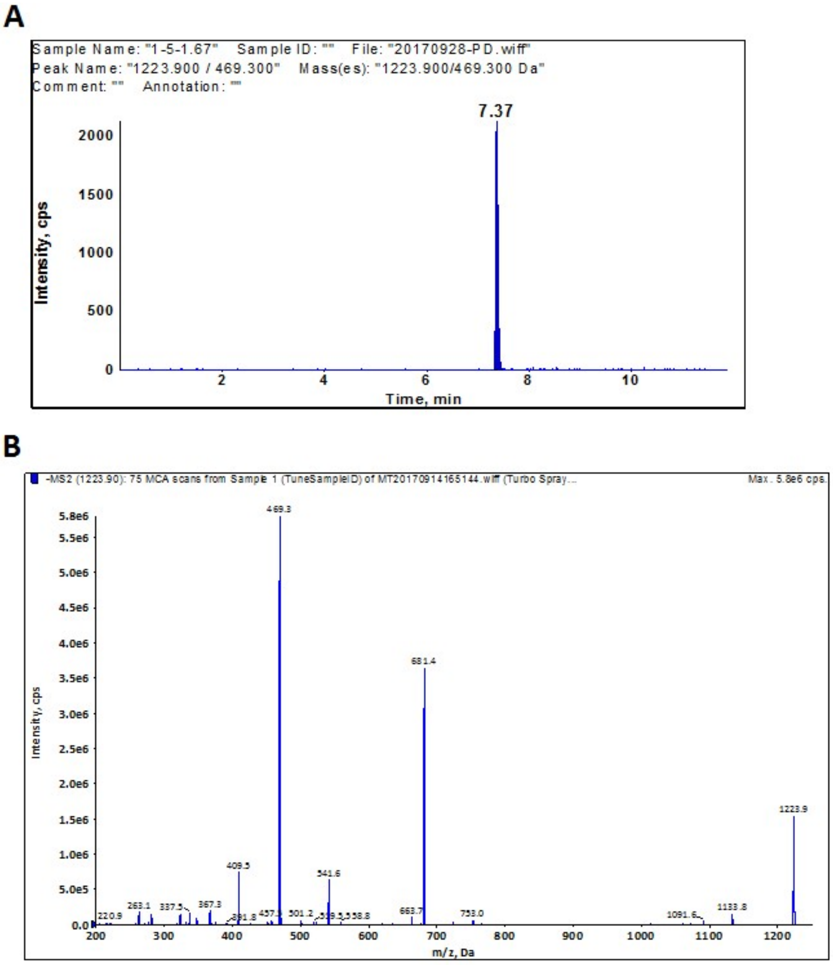

Figure S2. HPLC-MS/MS chromatography (**A**) and mass spectra (**B**) of PD in filtrate after direct interaction with PRRSV. Analysis of PD concentration was carried out using an Agilent 1200 series high-performance liquid chromatography (HPLC) system coupled with an Applied Biosystem API 4000 triple quadrupole mass spectrometer. Chromatographic separation was performed using an Agilent Zorbax SB-Aq C18 column (150 mm × 2.1 mm i.d., 3.5 μg/mL). The mass analysis was carried out under the negative electrospray ionization mode. The transitions of *m*/*z* 1223.9→469.3 was used for quantification, and of

m/*z* 1223.9→681.5 was used for identification. (**A**) Total ions chromatogram of PD. (**B**) ESI(-) mass spectra of PD.

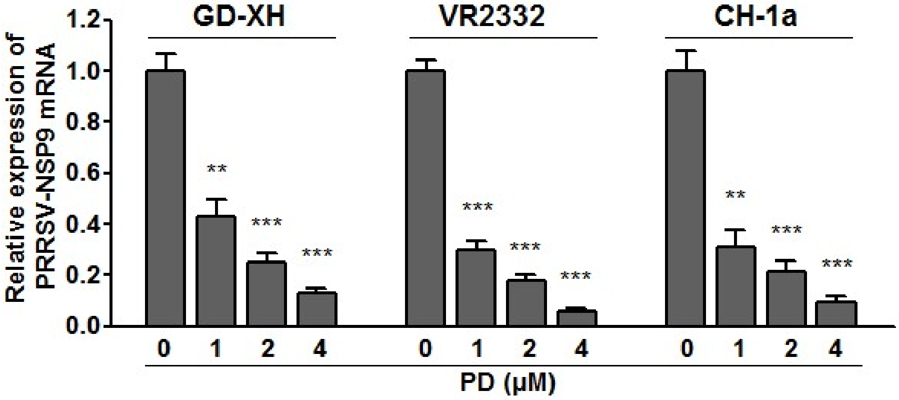

Figure S3. The anti-PRRSV activity of PD in PAM cultures is strain independent. PAMs grown in 6-well plates were infected with PRRSV GD-XH or VR2332 or CH-1a strain (0.5 MOI) for 2 h at 37°C and then treated with PD at various concentrations. Relative PRRSV NSP9 mRNA expression of PD treated groups to DMSO-treated control (0 μM PD) (set as 1) was analyzed using real-time RT-PCR at 24 h post PD treatment. *$p < 0.05$, **$p < 0.01$, and ***$p < 0.001$ compared to DMSO-treated control.
